# Combining disparate data sources for improved poverty prediction and mapping

**DOI:** 10.1073/pnas.1700319114

**Published:** 2017-10-31

**Authors:** Neeti Pokhriyal, Damien Christophe Jacques

**Affiliations:** ^a^Computer Science and Engineering, State University of New York, Buffalo, NY 14221;; ^b^Earth and Life Institute–Environment, Université Catholique de Louvain, 1348 Louvain-la-Neuve, Belgium

**Keywords:** poverty mapping, Gaussian process, mobile phone, remote sensing

## Abstract

More than 330 million people are still living in extreme poverty in Africa. Timely, accurate, and spatially fine-grained baseline data are essential to determining policy in favor of reducing poverty. The potential of “Big Data” to estimate socioeconomic factors in Africa has been proven. However, most current studies are limited to using a single data source. We propose a computational framework to accurately predict the Global Multidimensional Poverty Index (MPI) at a finest spatial granularity and coverage of 552 communes in Senegal using environmental data (related to food security, economic activity, and accessibility to facilities) and call data records (capturing individualistic, spatial, and temporal aspects of people). Our framework is based on Gaussian Process regression, a Bayesian learning technique, providing uncertainty associated with predictions. We perform model selection using elastic net regularization to prevent overfitting. Our results empirically prove the superior accuracy when using disparate data (Pearson correlation of 0.91). Our approach is used to accurately predict important dimensions of poverty: health, education, and standard of living (Pearson correlation of 0.84–0.86). All predictions are validated using deprivations calculated from census. Our approach can be used to generate poverty maps frequently, and its diagnostic nature is, likely, to assist policy makers in designing better interventions for poverty eradication.

## Significance

Spatially finest poverty maps are essential for improved diagnosis and policy planning, especially keeping in view the Sustainable Development Goals. “Big Data” sources like call data records and satellite imagery have shown promise in providing intercensal statistics. This study outlines a computational framework to efficiently combine disparate data sources, like environmental data, and mobile data, to provide more accurate predictions of poverty and its individual dimensions for finest spatial microregions in Senegal. These are validated using the concurrent census data.

More than 330 million people are still living in extreme poverty in Africa (1). Consequently, the goal to “eradicate extreme poverty for all people everywhere by 2030” tops the list of the 17 Sustainable Development Goals adopted by world leaders at the United Nations summit in September 2015. The lack of good-quality and fine-grained data to assess poverty regularly features in discussions of the development agenda for Africa (2, 3). Timely measurement and availability of data are vital in ending poverty.

Despite the nature of the strategies used to reduce poverty, governments and development agencies need a baseline depiction. Poverty maps provide such a spatial distribution of the socioeconomic deprivations and help policy makers assess the impact of interventions. For efficient targeting of policies at microregions and specific demographics, poverty maps should be made available at the finest administrative unit of planning. Also, these values should be disaggregated into individual dimensions of poverty, like deprivations in education, standard of living, health, and so forth (4).

Currently, the most reliable way to estimate poverty is through intensive socioeconomic household surveys. However, this approach is costly and time consuming and can only be realistically carried out for a small sample of households. The extrapolation of the local poverty estimation to a larger scale is traditionally done by exploiting links between census (wide area) and survey (smaller area coverage) data through small area estimation methods (5, 6). These techniques depend on the timely availability of census, which is typically collected every 10 y and whose analysis is delayed for poorer economies by years, making timely updates of poverty challenging.

Recently, there has been a growing interest in realizing the potential of “Big Data” to understand societal development in Africa. However, most current studies are limited to using single source datasets, such as mobile phone data (7) or satellite imagery (8). Since poverty is a complex phenomenon, understanding it using multiple lenses obtained from diverse datasets will help to chart more accurate maps for poverty.

Several studies highlight that significant spatial variation of poverty may be due to a variety of geographic factors, including agrometeorological conditions, accessibility and proximity to markets, access to land, and so forth (9, 10) (see Table S3). Earth Observation Satellites collect data on metrics such as nighttime lights, vegetation cover, and meteorological conditions. The unique features of such datasets are their global coverage, high revisit capability, and free availability. A complementary resource lies in Geographic Information Systems (GIS) analysis. In particular, proximity to important services (schools, hospitals) and density of infrastructure (such as roads) are all factors that might contribute to alleviating poverty (11).

**Table S3.** Brief review of poverty estimation methods based on environmental data

Ref.	Poverty variable	Model	Important variables	Main conclusions	Region
(49)	Daily consumption expenditure	Regression, correlation	Indoor air pollution (wood/charcoal use), access to clean water, no sanitation, diarrhea, outdoor air pollution (number of deaths from PM10)	Substantial variability across countries	Cambodia, Lao PDR, Vietnam
(50)	Per capita income	Regression	Mean road density, share in in internal revenue allotment, agrarian reform accomplishment rate, population growth, distance to major cities, mean elevation, percentage of slope with agricultural limitations, and mean annual rainfall	Spatial variation in poverty is mainly caused by disparities on access to road infrastructure	Philippines
(51)	Food expenditure	Regression and clustering	Proportion of irrigation land, average landholding sizes	Poverty maps show significant spatial clustering of poor and nonpoor areas	Sri Lanka
(11)	Per capita expenditure	Spatial regression	Slope, soil type, distance/travel time to public resources, elevation, type of land use, demographic variables	Increasing access to roads and improving soil conditions would result in decline in poverty	Kenya
(52)	Per capita expenditure	Regression	Distance to town, soil quality, slope	Poverty in the remote areas is linked to low agricultural potential and lack of market access	Vietnam
(53)	Household expenditure	Discriminant analysis	Distance to market, agro-climatic variables, diseases risk, livestock density	Satellite-derived variables tended to dominate the list of selected variables that determine poverty predictions	Uganda
(8)	Household consumption expenditure, asset wealth	Transfer learning (deep learning)	Roofing material, distance to urban areas	Interesting potential of machine learning method using limited training data	Nigeria, Tanzania, Uganda, Malawi, Rwanda
(54)	Household expenditure	Spatial regression, geographically weighted regression	Crop diversity, education, nonagricultural economic activities	Spatial nonstationarity of the relationship between poverty and its determinants	Malawi
(55)	Household income	Geographically weighted regression	Education, accessibility, and services	High poverty incidence that corresponds with ecologically depressed areas. However, other livelihood-influencing factors such as education, accessibility, and services are significantly correlated with poverty.	Bangladesh
(56)	Relative welfare (female literacy, land ownership, deprived class, and water source)	Random forests	Travel time to market towns, percentageof a village covered with woodland, and percentage of a village covered with winter crop	Satellite sensor data are strongly associated with aspects of rural welfare for an extensive region of a developing country	India

While satellite and GIS data are apt to observe and understand the availability of and access to natural resources and manmade structures, they lack information about population structure, especially the socioeconomic ties, cultural interactions, and micro- and macrobehavior that is essential to understanding poverty. One way to study societal interactions is provided by the widespread use of digital technologies (12). The Internet is still finding ground in sub-Saharan Africa. However, mobile phones are a prevalent technology, with adoption rates of more than 70%, even with 43% of population living in abject poverty (13). Such widespread use of mobile phones generates an unprecedented volume of data called call data records (CDRs). CDRs capture how, when, where, and with whom individuals communicate. These data, traditionally used by the telecommunication companies for billing purposes, capture both micro- and macropatterns of human interaction, while preserving the individual anonymity via spatial and temporal aggregation.

Poverty has traditionally been measured in one dimension, usually income or consumption, called income poverty. Another internationally comparable measure is the Global Multidimensional Poverty Index (MPI), which is used in this study. Global MPI is a composite of 10 indicators across three critical dimensions—education (years of schooling, school enrollment), health (malnutrition, child mortality), and standard of living conditions (see *Global MPI*). Throughout the paper, “poverty” refers to the Global MPI, and “dimensions” refers to education, health, and standard of living. MPI is calculated as a product of the incidence or headcount of poverty (H) and the average intensity (A) across the poor. H is the proportion of the population that is multidimensionally poor. A is the average proportion of indicators in which poor people are deprived.

**Fig. 1.**
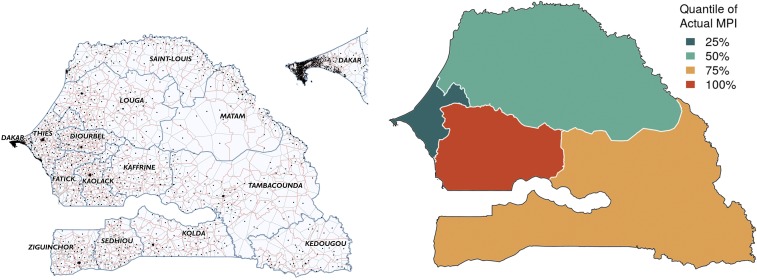
Details about the target country, Senegal. On the *Left* is a composite map of Senegal. Black dots depict the location of mobile towers (antennas). The Voronoi tessellation formed by these towers is shown in gray. The commune (which is the finest administrative unit in Senegal) boundaries are shown in red. There are 552 communes with 431 rural communes and 121 urban centers. The navy blue boundaries are those of regions, which are the coarsest administrative units in Senegal. There are 14 regions that are named in the map. On the *Right* is the current (2016) map of Global MPI for four divisions of the country (West, North, South, and Center).

**Fig. 2.**
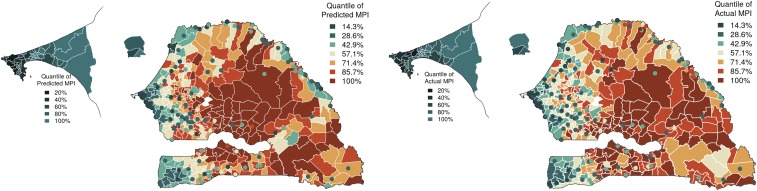
Quantiles of predicted (*Left*) and actual (*Right*) MPI at the commune level. The urban centers are depicted by small circles on the map. The communes in the Dakar and Thiès regions are shown enlarged.

The study focuses on Senegal, a sub-Saharan country that suffers from persistently high poverty. This study uses mobile phone data in the form of CDRs, and data related to food security (availability and access components), economic activity, and access to services are grouped together as environmental data (Table 1). The CDR variables capture not only the basic phone use statistics of a user but also the regularity, diversity, and spatiotemporal variability in the user’s mobile interactions. Tables S1 and S2 detail the variables extracted from CDR and environment data, respectively. The poverty maps are produced at the spatially finest level of policy planning, called “communes,” and validated at that level using the concurrent census data. Current poverty maps, based on Global MPI (see Fig. 1) and consumption-based measures (14), do not exist uniformly for all communes of Senegal. The map produced by our analysis is available for all 552 communes (see Fig. 2). Such maps can be generated frequently in between cycles of surveys and census, since CDR and environmental data are available at fine temporal granularity.

**Table 1.** Summary statistics and characteristics of the data used—CDRs, environment, census, and MPI

Summary statistics	CDRs	Environment data	Census	Poverty index
Timeline	January–December 2013	1960–2014	2013	2013
Number of total calls and text	11 billion	N/A	N/A	N/A
Number of unique individuals	9.54 M	N/A	1.4 M	N/A
Spatial granularity of available data	Antenna level (1666)	Vector data—100 m^−1^⋅km	Household level	Region level (14)
Cost incurred in data collection and preparation	Low/no cost (data exhaust)	Low/no cost (data exhaust)	US$29 million	Very high cost, and human expertise
Frequency of update of data	Real time	∼1 y	3–5 y	3–5 y

**Table S1.** Source, unit, and expected relationship to poverty of each environmental variable used in this study

Feature (no. of statistics)	Unit	Type of data	Endogeneity	Data sources	Expected relationship to poverty
Food security, availability					
Temperature—annual, annual range, diurnal range, warmest month, warmest quarter, coldest month, coldest quarter, wettest quarter, driest quarter, isothermality (11)	Degree Celsius	Ground	Exogenous	WorldClim database, 1960–1990 (45)	High temperature (+)
Precipitation—annual, wettest month, wettest quarter, driest month, driest quarter, warmest quarter, coldest quarter, coefficient of variation (8)	Millimeter	Ground	Exogenous	WorldClim database, 1960–1990 (45)	Low precipitation (+)
Elevation (1)	Meter	Remote sensing	Exogenous	CGIAR-SRTM data aggregated to 30 s (www.diva-gis.org/)	High elevation (+)
Slope (1)	Degree	Remote sensing	Exogenous	CGIAR-SRTM data aggregated to 30 s	High slope (+)
Soil type (14)	% of territory	Ground	Exogenous	Soil and Terrain Database for Senegal and the Gambia (version 1.0), scale 1:1 million (SOTER Senegal Gambia, www.isric.eu/projects/soter-senegal-and-gambia)	Poor agronomic soil (+)
NDVI (2)	—	Remote sensing	Endogenous	10-d temporal synthesis of 1 km SPOT-VEGETATION satellite images (2000–2013) (www.vgt.vito.be)	Low NDVI (in rural areas) (+)
Crop production (7)	Ton	Ground	Endogenous	Direction de Analyse, de la Prévision et des Statistiques Agricoles (DAPSA) 2000–2014 database (46)	Low production (in rural areas) (+)
Food security (access)					
Millet price (1)	CFA franc/kilogram	Ground	Endogenous	Modeling based on local supply and demand (47)	High millet price (+)
Proximity to urban centers, Market (1)	Kilometer	GIS	Endogenous	ANSD	Far from urban centers (+)
Proximity to main roads (1)	Kilometer	GIS	Endogenous	Open Street Map (www.openstreetmap.org)	Far from main road (+)
Economic activity					
Nighttime lights (2)		Remote sensing	Endogenous	Version 4 of the 2013 nighttime lights time series captured by the Operational Linescan System of the Defense Meteorological Satellite Program (stable lights)	Low density of of light (+)
Density of roads (1)	Kilometer	GIS	Endogenous	Open Street Map	Low density of roads (+)
Land cover					
Land cover (20)	% of territory	Remote sensing	Exogenous/endogenous	2005 1:100,000 scale Senegal Land Cover Map produced by the Global Land Cover Network (48) based on GlobCover 2005 map (33)	Urban areas (−), cropland (+), forest (+), grassland (+)
Access to facilities					
Proximity to school/university (1)	Kilometer	GIS	Endogenous	Open Street Map	Far from school/university (+)
Proximity to water tower (1)	Kilometer	GIS	Endogenous	Open Street Map	Far from water tower (+)
Proximity to hospital (1)	Kilometer	GIS	Endogenous	Open Street Map	Far from hospital (+)
Total	81				

**Table S2.** List of core features extracted for each individual from CDR data using the Bandicoot toolbox (31)

Features (no. of statistics)	Description
Regularity	
Interevent time (4)	The interevent time between two records of the user.
Diversity	
Number of contacts (2)	The number of contacts with whom the user interacted (call and text handled separately).
Entropy of contacts (2)	The entropy of the user’s contacts, both for call and text.
Balance of contacts (4)	The balance of interactions per contact. This feature is calculated—each for text and call. For every contact, the balance is the number of outgoing interactions divided by the total number of interactions (in + out).
Interactions per contact (4)	The number of interactions a user had with each of his or her contacts.
Percent pareto interactions (2)	The percentage of user’s contacts that account for 80% of his or her interactions.
Percent pareto durations (1)	The percentage of user’s contacts that account for 80% of his or her total time spend on the phone.
Active behavior	
Percent nocturnal (2)	The percentage of interactions the user had at night (call and text).
Percent initiated conversations (1)	The percentage of conversations that have been initiated by the user both for call and text.
Percent initiated interactions (1)	The percentage of calls initiated by the user.
Response delay (2)	The response delay of the user within a conversation (in seconds). This is calculated for text (SD and mean of the response delay).
Response rate (1)	The response rate of the user (between 0 and 1).
Basic phone use	
Active days (1)	The number of days during which the user was active.
Call duration (2)	The SD and the mean of the duration of user’s calls.
Number of interactions (6)	The number of interactions.
Ratio of text and call interactions (1)	This computes the ratio of the text and call interactions.
Spatial behavior	
Number of antennae (1)	The number of unique places visited.
Entropy of antennas (1)	The entropy of visited antennas.
Percent at home (1)	The percentage of interactions the user had while he or she was at home.
Radius of gyration (1)	Returns the radius of gyration, the equivalent distance of the mass from the center of gravity, for all visited places.
Frequent antennas (1)	The number of locations that accounts for 80% of the locations where the user was.
Churn rate (2)	The SD and mean of the frequency spent at every antenna each week.
Total	43

Features are grouped into categories based on prior research (29). These features are calculated for each month, so in total there are 43 × 12 = 516 features.

Our objective is to present a computational framework that integrates disparate data sources to accurately predict the Global MPI and its individual dimensions at the finest level of spatial granularity. This framework consists of models trained independently on each data source. Each source-specific model uses Gaussian process (GP) regression (GPR) (15) to infer poverty values. GP falls under the class of kernel methods, where the choice of different kernel functions enables one to learn different nonlinear relationships between the independent and target variables. Each GP-based model provides a probabilistic estimate of poverty for a given commune, including the mean and variance of the estimates. The variance provides a measure of uncertainty, which allows us to combine the predictions from the multiple data sources. An important advantage of this methodology is that the different data ecosystems need not share any data between them. The individual datasets remain private within their specific ecosystems, and only the output predictions and the associated variances are shared.

## Global MPI

Poverty has traditionally been measured in one dimension, usually income (or consumption)—also known as income poverty. Another internationally comparable measure is the Global MPI, which complements income poverty and is created from nationally representative Demographic and Health Surveys and Multiple Indicator Cluster Survey (DHS-MICS) (42). It was developed by OPHI and the United Nations Development Program. It is a composite of 10 indicators across three critical dimensions—education (years of schooling, school enrollment), health (malnutrition, child mortality), and living conditions (cooking fuel, sanitation, access to drinking water, electricity, floor, asset ownership).

MPI is defined as the percentage of people who are MPI poor (H, headcount of poverty) multiplied by the average intensity of MPI poverty across the poor (A, intensity of poverty). The MPI data for Senegal, used in this study, were downloaded from www.ophi.org.uk/wp-content/uploads/Senegal-2013.pdf.

MPI is robust to decomposition within relevant subgroups of populations, like urban vs. rural, geographic regions (districts/provinces/states), and gender, so that targeted policies can be planned for specific demographics. Countries can also adapt the multidimensional poverty approach to select different indicators and/or update weights that align better with their nation’s poverty measure. Countries, like Mexico, Colombia, and Chile, have implemented their own version of national MPI using additional dimensions than MPI, such as employment and social protection, when data are available (www.mppn.org/).

The MPI has some limitations. Though it has been defined from available variables in global surveys (DHS-MICS), some of the potential dimensions of poverty (like gender, income, employment) are not directly incorporated. However, due to the wide availability of these surveys, Global MPI can easily be estimated in more than 100 countries covering 5.2 billion people (42). Consequently, it represents a benchmark index, more interesting than the single dimension poverty line, for replication of this study in another country.

## Results

### GP Model for Predicting Poverty from a Single Data Source.

To predict poverty for a commune from a single data source (CDR or environment), the following model is assumed:

$$y_{i}=\beta^{⊤}{\text{𝐱}}_{i}+f\left({\text{𝐱}}_{i}\right)+\epsilon$$ [1]

where $y_{i}$ is the target poverty value and ${\text{𝐱}}_{i}$ is a vector of independent variables derived from the particular data source for the *i*th commune. The first term is a linear combination of the independent variables. The function f() models the nonlinear relationship between $y_{i}$ and ${\text{𝐱}}_{i}$. The residual term, ϵ, models the remaining unexplained noise and is modeled as a zero-mean Gaussian random variable—that is, $\epsilon ∼N\left(0,\sigma_{n}^{2}\right)$.

**Fig. S6.**
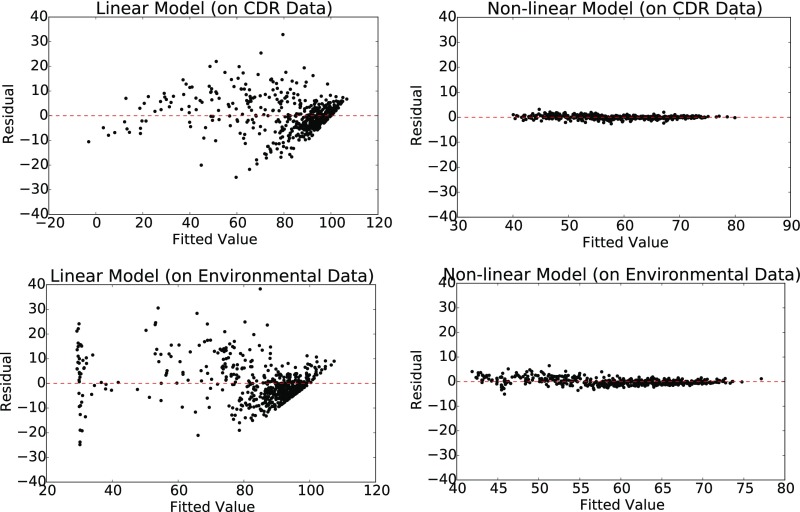
Residual vs. fit plots to predict incidence of poverty (H) using CDR (*Top*) and environmental (*Bottom*) data. (*Left*) Linear (elastic net regression). (*Right*) Nonlinear (GPR). Linear model fits indicate nonlinearity in the data. The residuals for GPR are normally distributed. Shapiro–Wilk test statistic: CDR, 0.97 (*P* value $<10^{-9}$); environmental, 0.95 (*P*-value $<10^{-9}$).

Without the nonlinear term, f() in Eq. **1**, the model is equivalent to ordinary linear regression. However, a linear model is not rich enough to capture the relationships between the target and the independent variables (see Fig. S6), thus motivating the need for a nonlinear term.

Instead of assuming a fixed parametric form for f(), we adopt a nonparametric approach, by assuming a GP prior on f(). The generative process thus becomes:

f(𝐱)∼GP(m(𝐱),k(𝐱,𝐱′)) [2]

$$y_{i}∼N\left(\beta^{⊤}{\text{𝐱}}_{i}+f\left({\text{𝐱}}_{i}\right),\sigma_{n}^{2}\right),\forall i$$ [3]

A GP is a stochastic process, indexed by $\text{𝐱}\in {\mathbb{R}}^{d}$. Any finite sample generated from it is jointly multivariate normal (15). m(𝐱) is the mean of f(𝐱) and k(𝐱,x’) is a kernel function that defines the covariance between any two evaluations of f(𝐱)—that is, m(𝐱)=𝔼[f(𝐱)], and k(𝐱,𝐱′)=𝔼[(f(𝐱)−m(𝐱))(f(𝐱′)−m(𝐱′))]. For model simplicity, we assume that m(𝐱)=0, which is a standard practice in GP-based methods (15).

Given a training set of examples, $D={\left\{{\text{𝐱}}_{i},y_{i}\right\}}_{i=1}^{N}$, the GP prior on f(), and other terms in Eq. **1**, the posterior distribution of $y_{*}$ (for an unseen input vector, ${\text{𝐱}}_{*}$) is a Gaussian distribution, with the following mean and variance (see *GP Regression Model* for details):

$${\bar{y}}_{*}:=𝔼\left[y_{*}\right]=\beta^{⊤}\text{𝐱}+{\text{𝐤}}^{⊤}{\left(K+\sigma_{n}^{2}I\right)}^{-1}\text{𝐲}$$ [4]

$$\sigma_{*}^{2}:=\text{var}\left[y_{*}\right]=k_{*}-{\text{𝐤}}^{⊤}{\left(K+\sigma_{n}^{2}I\right)}^{-1}\text{𝐤}+\sigma_{n}^{2}$$ [5]

Here, $\text{𝐲}={\left[y_{1},y_{2},\ldots \right]}^{⊤}$, and K is a matrix that contains the kernel function evaluation on each pair of training inputs—that is, $K\left[i,j\right]=k\left({\text{𝐱}}_{i},{\text{𝐱}}_{j}\right)$—and **k** is a vector of the kernel computation between each training input and the test input—that is, $\text{𝐤}\left[i\right]=k\left({\text{𝐱}}_{*},{\text{𝐱}}_{i}\right)$, $k_{*}=k\left({\text{𝐱}}_{*},{\text{𝐱}}_{*}\right)$—and I is an identity matrix.

### Choice of Kernel Function.

The role of the kernel function is to specify how the function values f(𝐱) and f(𝐱′) vary as the function of their corresponding inputs **x** and 𝐱′. We use the following kernel function:

$$k\left(\text{𝐱},\text{𝐱}\prime \right)=\sigma_{f}^{2}\exp\left(-\frac{{∥\text{𝐱}-\text{𝐱}\prime ∥}^{2}}{2\ell^{2}}\right)\exp\left(-\frac{{∥{\text{𝐱}}_{s}-{\text{𝐱}\prime }_{s}∥}^{2}}{2\ell_{s}^{2}}\right)$$ [6]

where ${\text{𝐱}}_{s}$ and ${\text{𝐱}\prime }_{s}$ are the spatial coordinates (latitude, longitude) of the commune centers corresponding to **x** and 𝐱′, respectively. The first exponent term captures nonlinear dependencies in the feature space. The second exponent term plays the same role, but in the geographic space and models, the spatial autocorrelation is a continuous function, which is same as Kriging, a widely used method in geostatistics (16). The parameter $\sigma_{f}^{2}$ is the variance of the stochastic process f, l is the process length scale for the feature space part, and $l_{s}$ is the process length scale for the spatial part.

The quantities $\beta ,\ell ,\ell_{s},\sigma_{n}^{2}$, and $\sigma_{f}^{2}$ are estimated by maximizing the marginalized log-likelihood of the training data, as discussed in *Materials and Methods*. To remove the effect of spurious features, we couple the GP model with elastic net regularization (17) during the model learning phase. This allows for automatic relevant feature selection and learning a parsimonious model that improves interpretability.

### Combining Source-Specific Models.

To predict poverty for a commune, we use two independently trained models specified in Eq. **1**, corresponding to the two data sources of CDRs and environmental data. Each model produces a posterior Gaussian distribution, denoted by $y_{ic}∼N\left({\bar{y}}_{ic},\sigma_{ic}^{2}\right)$ and $y_{ie}∼N\left({\bar{y}}_{ie},\sigma_{ie}^{2}\right)$ for the CDR and environmental data, respectively. The combined poverty estimate, $y_{i}$, is assumed to be a mixture distribution consisting of two Gaussians, defined above, and the mixing weights defined as:

$$w_{ic}=\frac{\frac{1}{\sigma_{ic}^{2}}}{\frac{1}{\sigma_{ic}^{2}}+\frac{1}{\sigma_{ie}^{2}}},w_{ie}=\frac{\frac{1}{\sigma_{ie}^{2}}}{\frac{1}{\sigma_{ic}^{2}}+\frac{1}{\sigma_{ie}^{2}}}$$ [7]

The weights assign greater importance to the source that provides a smaller predictive variance, signifying higher confidence in the prediction for the particular commune. The mean and the variance for the combined poverty estimate are (see *Estimating Moments of a Mixture Distribution*):

$$𝔼\left[y_{i}\right]=w_{ic}{\bar{y}}_{ic}+w_{ie}{\bar{y}}_{ie}\text{var}\left[y_{i}\right]=w_{ic}\sigma_{ic}^{2}+w_{ie}\sigma_{ie}^{2}+w_{ic}w_{ie}{\left({\bar{y}}_{ic}-{\bar{y}}_{ie}\right)}^{2}$$ [8]

### Predicted MPI Poverty Values.

The predicted map of MPI for microregions—that is, 552 communes of Senegal—is depicted in Fig. 2, *Left*. Compared with the current poverty map in Fig. 1, our map highlights heterogeneity in the existence of poverty within each macroregion. The communes toward the interior of the country have more poverty compared with the rest. The west regions, containing the capital city Dakar, and communes neighboring the coastal boundary are less poor than the rest of the country. Of special interest is the spatially large division in the south, consisting of the regions of Tambacounda, Kedougou, and Kolda, which are depicted as one color on the current map in Fig. 1 but have communes of varying poverty values spread throughout. Interestingly, the communes in the Kedougou region in the extreme southeast corner of Senegal are predicted as wealthier than other communes within the region. The communes in the region of Ziguinchor, in the southwest corner, are wealthier compared with other communes in the south. This is attributed to the fact that Ziguinchor is the second largest city in Senegal, with the economic advantage of being a port and a tourist center.

The 121 urban centers are shown as small circles on the map and, in general, have less poverty values compared with rural communes. The population in urban centers is generally richer than the population living in adjacent rural communes. This is true even for very poor communes of Senegal in the regions of Kaffrine and Tambacounda in the center, for which the contrast is even higher. The urban centers bordering with the neighboring country Mauritania, in the northeast, are wealthier; this could be attributed to the economy of the Senegal river basin and to cross-border trade. The predominantly urban areas in Dakar are shown enlarged in the map. All communes in Dakar are more well-off than the rest of Senegal because of the concentration of economic activity over the years.

A quantitative validation of the predictions is provided against commune-level poverty values estimated from census data (see Fig. 2, *Right*) using cross-validation (CV) procedures (details in *Materials and Methods*). A standard CV is often performed to ensure that the model generalizes to out-of-sample data. We performed a standard 10-fold CV, where the data are randomly split into 10-folds. Each time, ninefolds are used for training, and singlefold is used for evaluation, meaning we randomly assign 90% of communes to the training set and evaluate the remaining 10% of communes. This procedure is repeated 250 times to provide a robust assessment of the variability of model parameters and prediction statistics. Using standard CV, the model gives a Pearson’s correlation of 0.94, with a *P* value of <0.0001. Though training and evaluation data are selected randomly, the above-described method of validation may prove to be insufficient, as the poverty deprivations tend to be spatially correlated. Thus, a model may appear to perform well when evaluated this way, even though it may have poor extrapolation power in the spatial sense. The above results are provided for comparison.

To measure the extrapolation ability of the model to spatial areas that were not represented in the training data, we use a spatial CV procedure (18) (details in *Materials and Methods*). Here, the training and evaluation sets are sampled from geographically distinct regions ensuring that the model is tested rigorously. The experiments were repeated 250 times with random samples of training and evaluation sets, while ensuring that all communes are represented in the evaluation. We report Pearson’s and Spearman’s correlations, and rms error (RMSE), averaged over the multiple CV runs. The predictions in Fig. 2, *Left* have a spatially cross-validated Pearson’s correlation of 0.91 and rank correlation of 0.87, with *P* values less than $10^{-20}$ for both tests, indicating strong significance. This emphasizes the efficacy of our model in predicting poverty values accurately at the finest spatial granularity, using multisource data.

As a comparative study of how our model performs using multisource and single-source data, we experimented with three datasets—Multisource, CDR, and Environment—to predict H, A, and MPI at the commune level (see Table 2). We report highly accurate results for all three targets (H, A, and MPI). Rank correlations are preserved, as we report a Spearman’s correlation of 0.85 for both H and A. The values of Pearson’s *r* correlation are much higher than rank correlation, across all prediction tasks, indicating the linear correspondence of the poverty values with the predicted ones. We report significantly low *P* values ($<10^{-34}$) for spatial CV compared with standard CV, signifying more stable performance. For detailed results, see Table S5. Table 2 shows that combining multiple data sources (CDRs and environmental data) results in a consistent improvement of accuracy over using the individual data sources. The improvement is more pronounced in detailed results for all of the indicators of poverty and given in Table S5.

**Table 2.** Spatially cross-validated results of the predictions of MPI, headcount of poverty (H), and intensity of poverty (A), along with the individual indicators for poverty given by our model using disparate datasets

	Multisource data			CDR			Environment		
Poverty indicators and dimensions	Corr.	Rank corr.	RMSE	Corr.	Rank corr.	RMSE	Corr.	Rank corr.	RMSE
MPI	0.91 (0.06)	0.88 (0.06)	0.08 (0.01)	0.89 (0.07)	0.86 (0.07)	0.08 (0.01)	0.84 (0.09)	0.80 (0.10)	0.10 (0.02)
H	0.91 (0.07)	0.85 (0.08)	10.79 (3.96)	0.90 (0.08)	0.84 (0.08)	10.76 (2.60)	0.83 (0.11)	0.75 (0.11)	13.65 (4.86)
A	0.86 (0.05)	0.85 (0.07)	4.71 (0.96)	0.83 (0.07)	0.82 (0.08)	4.98 (1.14)	0.81 (0.07)	0.79 (0.08)	5.36 (0.75)
Education	0.86 (0.05)	0.84 (0.05)	11.84 (1.88)	0.82 (0.05)	0.81 (0.07)	13.08 (1.68)	0.76 (0.07)	0.74 (0.07)	14.98 (3.03)
Health	0.49 (0.15)	0.50 (0.16)	12.76 (2.12)	0.50 (0.12)	0.52 (0.12)	12.91 (1.92)	0.36 (0.23)	0.35 (0.23)	13.91 (2.32)
Standard of living	0.83 (0.11)	0.75 (0.13)	14.82 (3.92)	0.81 (0.11)	0.74 (0.11)	15.24 (3.45)	0.73 (0.18)	0.64 (0.20)	17.88 (4.50)

The results are compared when single source data are available. Corr., Pearson’s r correlation; rank corr., Spearman’s rank correlation; RMSE, rms error. For both types of correlations, all P values were less than 10^−20^. An SD associated with the multiple runs for each measurement is reported within parentheses.

**Table S4.** Brief review of poverty estimation methods based on CDR data

Ref.	Data source	Model (number of features)	Sample size	Time period	Results, Pearson’s *r*	Spatial resolution of validation	Poverty measure	Region
(7)	CDR and phone survey	Linear regression (5,088)	1.5 M (CDR) + 856 (survey)	9 mo	0.68	492 DHS clusters	DHS composite wealth index	Rwanda
(23)	CDR	Support vector machine (279)	500 K	6 mo	0.80	—	Socioeconomic levels	Urban area in a Latin American city
(57)	CDR	Linear regression (OLS) (5)	5 M and 928 K	20 wk and 6 wk	—	11 subprefecture level (CIV)	IMF poverty rate	Cote d’Ivoire and anonymous region B
(58)	CDR	Linear regression (33)	9 M and 150 K	12 mo	0.82	14 regions in Senegal	MPI (OPHI)	Senegal

**Table S5.** Spatially-cross validated results of the predictions of MPI, incidence of poverty (H), and intensity of poverty (A), along with the individual indicators for poverty given by our model using disparate datasets

	Multisource data			CDR			Environment			Concatenated		
Poverty indicator	Corr.	Rank corr.	RMSE	Corr.	Rank corr.	RMSE	Corr.	Rank corr.	RMSE	Corr.	Rank corr.	RMSE
MPI	0.91 (0.06)	0.88 (0.06)	0.08 (0.01)	0.89 (0.07)	0.86 (0.07)	0.08 (0.01)	0.84 (0.09)	0.80 (0.10)	0.10 (0.02)	0.90 (0.06)	0.85 (0.07)	0.10 (0.02)
H	0.91 (0.07)	0.85 (0.08)	10.79 (3.96)	0.90 (0.08)	0.84 (0.08)	10.76 (2.60)	0.83 (0.11)	0.75 (0.11)	13.65 (4.86)	0.90 (0.07)	0.83 (0.08)	11.34 (3.87)
A	0.86 (0.05)	0.85 (0.07)	04.71 (0.96)	0.83 (0.07)	0.82 (0.08)	04.98 (1.14)	0.81 (0.07)	0.79 (0.08)	05.36 (0.75)	0.84 (0.07)	0.82 (0.08)	5.52 (1.40)
Individual indicators of poverty												
*Education*												
Years of schooling	0.85 (0.04)	0.85 (0.04)	12.00 (1.21)	0.81 (0.05)	0.80 (0.06)	13.30 (1.55)	0.76 (0.07)	0.75 (0.08)	15.42 (2.48)	00.85 (0.04)	0.84 (0.04)	12.06 (01.01)
School attendance	0.86 (0.05)	0.83 (0.06)	11.68 (1.83)	0.82 (0.07)	0.81 (0.07)	12.85 (1.73)	0.75 (0.09)	0.72 (0.09)	14.54 (3.06)	0.85 (0.05)	0.83 (0.06)	11.60 (2.05)
*Health*												
Child mortality	0.45 (0.15)	0.46 (0.16)	10.91 (0.58)	0.45 (0.13)	0.48 (0.13)	11.32 (00.73)	0.34 (0.19)	0.33 (0.21)	11.54 (0.65)	0.45 (0.14)	0.45 (0.16)	10.85 (0.49)
Nutrition	0.52 (0.15)	0.53 (0.15)	14.61 (3.65)	0.54 (0.11)	0.55 (0.11)	14.49 (3.10)	0.38 (0.26)	0.37 (0.25)	16.28 (3.99)	0.47 (0.21)	0.46 (0.22)	15.33 (4.24)
*Standard of living*												
Cooking fuel	0.86 (0.14)	0.70 (0.18)	13.82 (8.76)	0.83 (0.14)	0.68 (0.16)	12.98 (7.00)	0.76 (0.20)	0.58 (0.25)	16.49 (8.78)	0.86 (0.13)	0.70 (0.18)	15.56 (9.19)
Sanitation	0.79 (0.17)	0.70 (0.18)	16.99 (3.42)	0.74 (0.17)	0.69 (0.17)	18.05 (3.14)	0.72 (0.22)	0.61 (0.26)	18.64 (4.33)	0.77 (0.20)	0.66 (0.23)	18.69 (3.91)
Water	0.75 (0.14)	0.72 (0.14)	14.60 (3.22)	0.74 (0.13)	0.71 (0.12)	14.70 (2.98)	0.67 (0.20)	0.61 (0.21)	16.97 (3.25)	0.68 (0.21)	0.62 (0.22)	17.15 (3.20)
Electricity	0.88 (0.04)	0.84 (0.07)	15.09 (0.98)	0.86 (0.04)	0.83 (0.06)	16.67 (1.25)	0.79 (0.10)	0.72 (0.13)	20.27 (1.72)	0.84 (0.05)	0.80 (0.09)	18.61 (1.65)
Floor	0.78 (0.15)	0.68 (0.14)	15.79 (5.79)	0.79 (0.13)	0.70 (0.12)	15.24 (4.93)	0.64 (0.24)	0.54 (0.23)	17.87 (6.22)	0.74 (0.19)	0.63 (0.16)	16.58 (5.81)
Asset ownership	0.89 (0.04)	0.86 (0.05)	12.61 (1.33)	0.87 (0.04)	0.85 (0.04)	13.81 (1.20)	0.80 (0.11)	0.75 (0.11)	17.05 (2.69)	0.85 (0.05)	0.82 (0.06)	15.37 (1.48)

The results are compared with models learned on single source and on concatenated feature space. Corr., Pearson’s *r* correlation; rank corr., Spearman’s rank correlation; RMSE, root mean square error. For both types of correlations, all *p* values were less than 10^−20^. An SD associated with the multiple runs for each measurement is reported within parentheses.

**Fig. 3.**
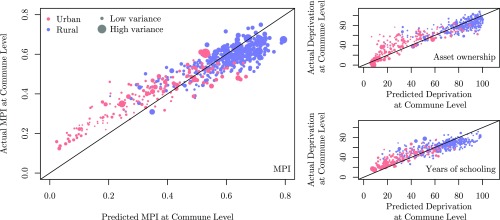
Predictive power of the Gaussian process model. *Left* denotes the comparison of actual and predicted MPI values for all communes and urban areas of Senegal. The rural and urban areas are differentiated using blue and red colors, respectively. The size of the circle denotes the variance of the MPI prediction for that commune. *Top Right* shows how the actual and predicted values compare for asset ownership, while *Bottom Right* shows the comparison for years of schooling.

**Fig. S5.**
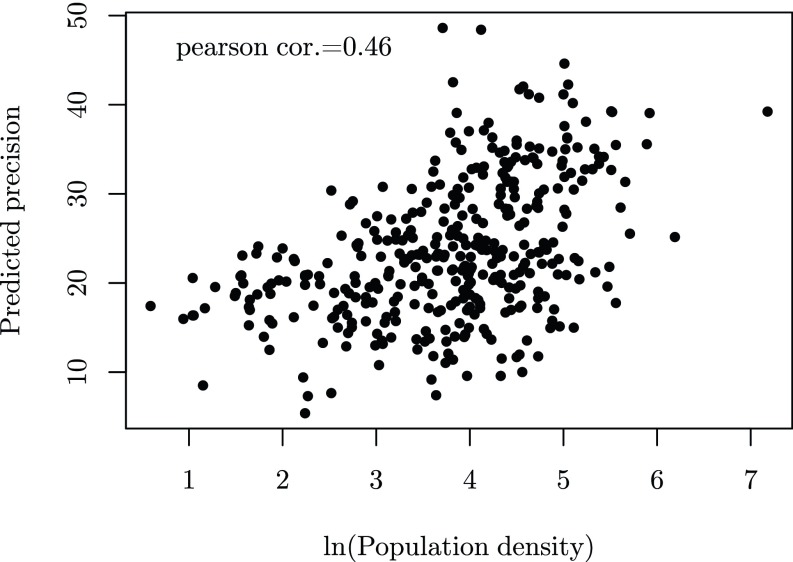
Relationship between precision of estimates of poverty and the population density of each commune.

Fig. 3, *Left* plots the relationship between MPI values predicted by our model and those estimated from census. We observe a linear relationship, in general, for MPI, with lower values for urban areas (shown in red) and higher values for rural areas (shown in blue). Predominantly urban communes of Dakar and a few urban centers are underestimated for poverty (i.e., they are predicted richer than they are). Likewise, there are very few rural communes, where poverty is overestimated. We also observe that for communes with lower population densities, the predicted variance is comparatively higher than it is for communes with higher densities, signifying that lesser numbers of data points in the vicinity of a given commune contribute to its higher variance (see Fig. S5).

### Predicted Values for the Dimensions of Poverty.

Global MPI consists of 10 individual deprivation indicators grouped along three dimensions: (*i*) education (indicators—years of schooling and school attendance), (*ii*) health (indicators—child mortality and nutrition), and (*iii*) standard of living (indicators—cooking fuel, sanitation, access to drinking water, electricity, and floor and asset ownership). Each individual deprivation indicator is taken as the target of our model, and the averaged spatially cross-validated results, along the three dimensions, are reported in Table 2. Detailed results for each of the 10 indicators are given in Table S5.

Referring to Table S5, we note that the accuracy of the model is high for some deprivations and good for most deprivations. All deprivations are better predicted using CDR data, probably because they characterize the individual behavior while environmental data depict conditions that might have an influence on poverty (see Tables S1 and S2). Fig. 3, *Top Right* compares our predictions for asset ownership with those estimated from the census. Rural communes depicted (by blue) are clustered closely toward high deprivation. The urban areas have, generally, lower deprivation than rural areas, though it is spread out.

Indicators related to education—years of schooling and school attendance—are predicted well, because use of short message service (SMS) is indicative of literacy (19). The environmental data also perform well, because they capture the distance to schools, main roads, and urban centers, all of which facilitate access to educational attainment. Fig. 3, *Bottom Right* shows that all areas of Senegal are deprived in education, as the rural (in blue) and urban (in red) points are spread evenly on the plot. However, rural areas tend to dominate at the very high deprivation index, while very low deprivation areas are urban.

The model performs poorly for the indicators within the health dimension—that is, child mortality and nutrition. This is attributed to the fact that our data are not representative of the children population, and thus, the features extracted from CDR data do not capture this deprivation. A similar inference can be drawn for poorer correlations for nutrition. Moreover, the validation of deprivation values computed from the census for nutrition indicators are based on two hunger-related questions, as detailed nutritional information is not available to us (see Table S7 for details).

**Table S7.** A summary of poverty indicators and associated deprivations, with emphasis on how our methodology calculates them using the RGPHAE census data, keeping in view the OPHI guidelines

Poverty indicators	Deprivation standards of a household used by OPHI for MPI calculation	RGPHAE census questionnaire response used by our methodology for MPI calculation
Health		
Child mortality	At least one child has died	About living and deceased children in the household
Nutrition	Any member is undernourished	About going hunger at night for the past few months
Education		
School attendance	Any school-aged child is not attending school up to grade 8	About school-aged currently not in school
Years of schooling	No member who has completed at least 5 y of education	About higher schooling of any member
Standard of living		
Cooking fuel	Uses solid fuels for cooking	Household does not use electricity or natural gas for cooking
Electricity	No access to electricity	No electricity or generator
Sanitation	No access to adequate sanitation or if it is shared	Household has no sewer connection or pit
Drinking water	No access to safe drinking water	No water tap in household
Flooring	Has dirt/earth/dung floor	Household has dirt/earth/dung floor
Assets	Has only one small asset (radio, TV, refrigerator, phone, bicycle, motorbike) and it has no car	Household has one asset (radio, TV, refrigerator, phone, bicycle, motorbike) and it has no car

### Dimensions of Poverty—Interpretation of Weights.

Figs. S2 and S3 display the features deemed important by our model for the environment and CDR data, respectively. The important features are those for which the corresponding entries in the coefficient vector, β, are high in magnitude. We ignore child mortality and nutrition, as our model does not perform very accurately for these two indicators. The following interpretations are given for information purposes. These are, by no means, indicators of causality.

**Fig. S1.**
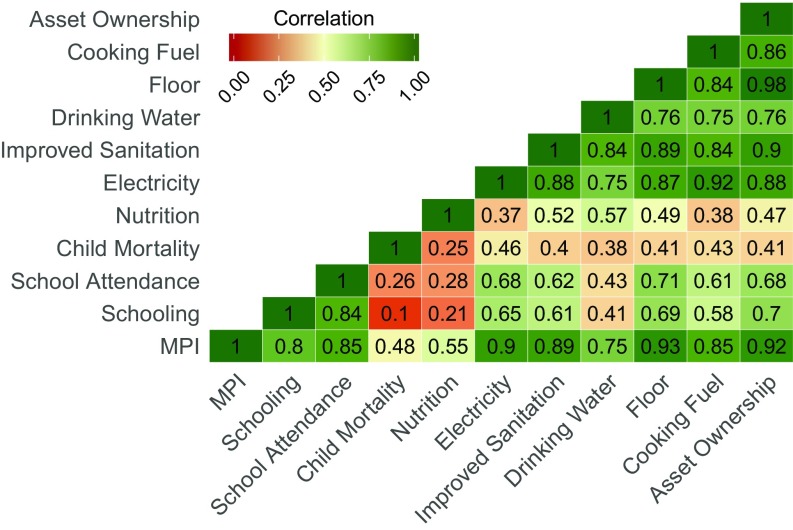
Spearman correlation matrix between individual deprivations, H (headcount of poverty), A (intensity of poverty), and MPI at the commune level.

**Fig. S2.**
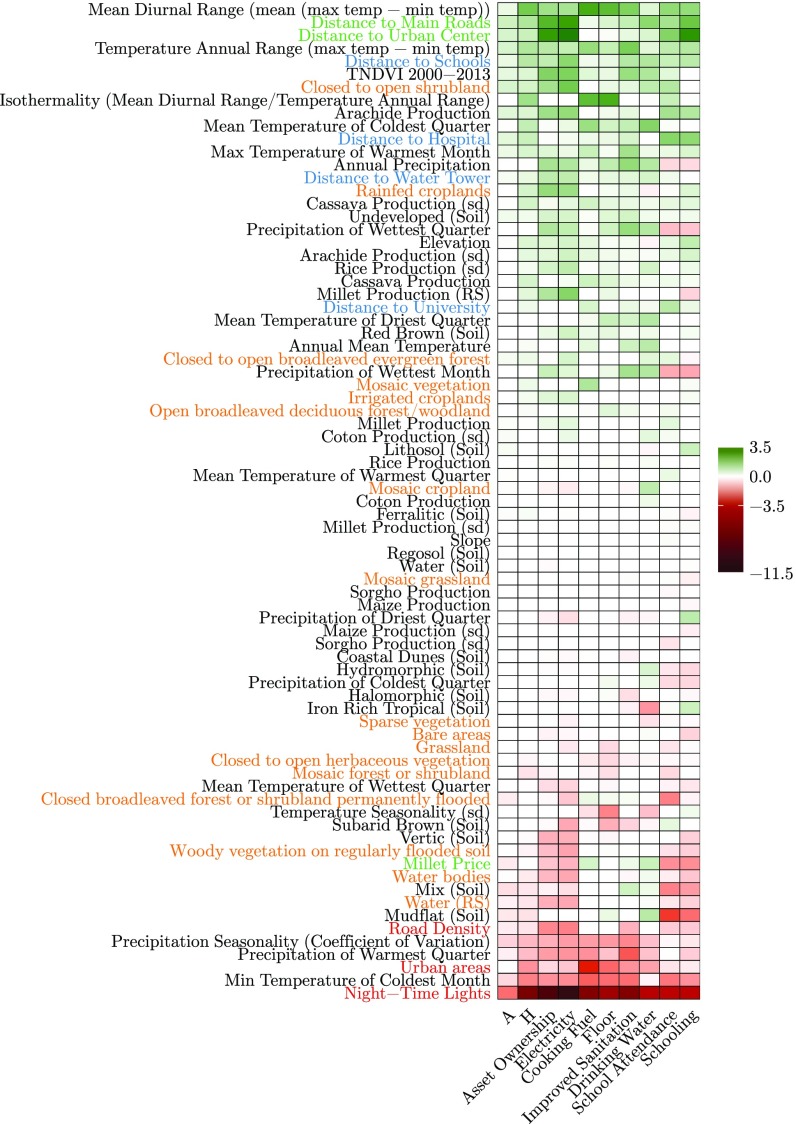
Visualization of selected features using elastic net regularization on environmental data for prediction of selected deprivations. The rows represent the features, which are ranked according to their weights from positive (marked green) to negative (marked red). Different features groups are color-coded. Features related to food availability are given in black color, whereas those related to food accessibility are colored green. The land cover features are colored yellow, and the features detailing economic activity are in red color. Finally, features depicting access to services are shown in blue. The cells in white were given 0 weights by our model.

**Fig. S3.**
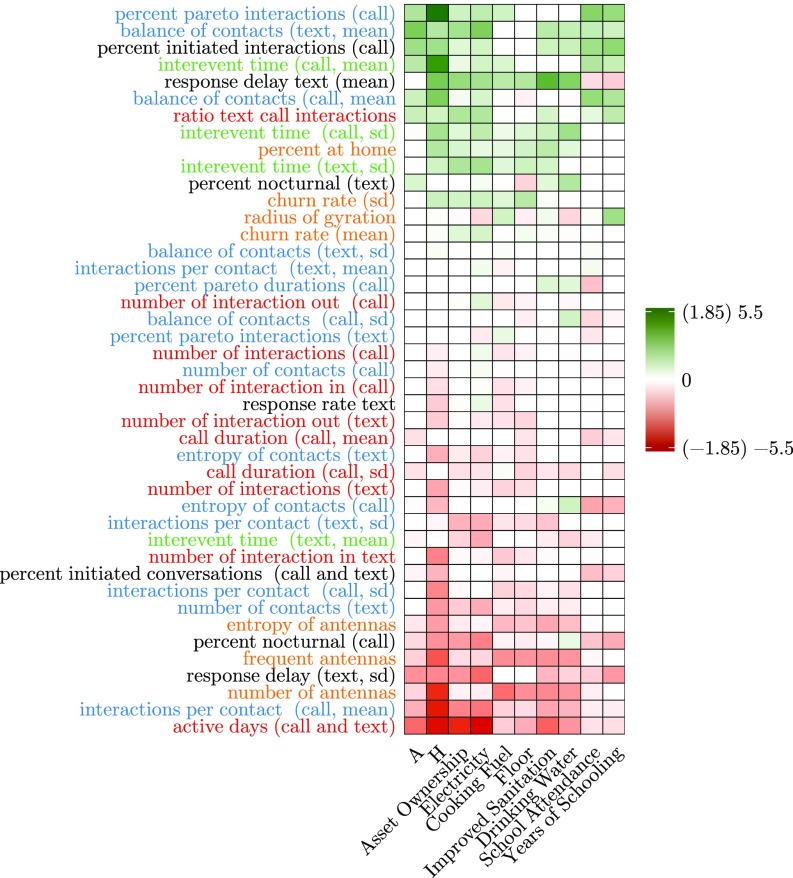
Visualization of selected features using elastic net regularization on CDR data for prediction of selected deprivations. The rows represent features, which are ranked according to their weights from positive (marked green) to negative (marked red). The columns are the various deprivations. The feature groups are color-coded. Features related to diversity features are colored blue. Those related to spatial aspects are colored yellow. The features related to active behavior are marked in black. The features related to basic phone use are in red, and those related to regularity are in green. The cells in white were given 0 weights by our model. Legend in parentheses corresponds to the different variation in weights. H and A weights vary between 1.85 and −1.85, and for others the weights vary between 5.5 and −5.5.

Referring to Fig. S2, nighttime lights appear to be the most important feature regardless of the predicted dimensions, conforming to the current research (8, 20). Nighttime lights show a strong correlation with MPI (Spearman correlation of −0.66). Urban areas and road density, two other important indicators of economic activity, are relevant but to a lesser extent. Even though the coefficient values of each dimension are not directly comparable, since each dimension was taken as a separate target, it is interesting to note that the weights of nighttime lights intensity for electricity and asset ownership deprivation are the highest. This result confirms previous findings (21) that access to electricity is correlated with nighttime lights (Spearman correlation of −0.67). Additional observations regarding water deprivation, food security (access component), and climate are given in *Interpretation of Weights—Along the Dimensions of Poverty*.

A similar analysis for the CDR features reveals several interesting insights regarding the relationship between poverty and the individual characteristics captured in CDR features. While we considered CDR features for each month individually, for the ease of visualization (see Fig. S3), we average the monthly values of the weights associated with each feature.

Here we discuss the CDR features that were selected by the model as the strongest predictors for the various targets. These features are listed in Table S6. One of the strongest negative predictors for most of the targets is the number of active days (for call and text), which characterizes that individuals in wealthier communes have monetary resources to recharge their phone and make/receive calls. The ratio of calls vs. text shows the preference for calls and emerges as an important factor to predict education-based deprivations. The feature “interevent time call” measures the irregularity in responding to calls/text and emerges as a positive predictor for deprivations. Features that indicate diversity in communication, such as entropy of contacts and interactions per contact (call and text), report a negative relationship to poverty. These results confirm previous findings (7, 22, 23) that diversity of an individual’s relationships is positively correlated with his or her economic wellbeing. However, for features such as percent pareto interactions and balance of contacts, which are proportional to an individual’s diversity in communication, we report a positive relationship with poverty. This counterintuitive relationship needs to be further studied in the context of telecommunication patterns in Senegal.

**Table S6.** List of the important features chosen by our model to predict each of H, A, schooling, school attendance, cooking fuel, sanitation, water, electricity, floor, and assets

Feature type	H	A	Schooling	School attendance	Cooking fuel	Sanitation	Water	Electricity	Floor	Assets
Basic										
Active days call and text	–	–			–	–	–	–	–	–
Ratio of call/text interactions			+			+		+		+
Number of interactions in text					–					
Regularity										
Interevent time call, mean	+	+	+	+						
Interevent time call/text, SD						+	+	+	+	+
Diversity										
Balance of contacts text, mean		+	+	+		+	+	+		+
Percent pareto interactions call	+	+	+	+	+			+		+
Interactions per contact call, mean	–	–			–	–	–	–		–
Entropy of contacts call			–	–				–		
Active										
Response delay text, mean	+		–		+	+	+	+	+	+
Response delay text, SD		–	–	–		–	–	–		–
Percent initiated interactions, call		+	+	+		+	+			
Percent initiated conversations, call and text			–	–						
Spatial										
Frequent antennas	–				–	–	–		–	
Number of antennas	–				–	–	–		–	
Radius of gyration			+		+					
Entropy of antennas					–	–	–		–	
										

The features having positive relationships with the various deprivations are marked as + in the cell corresponding to the feature name and the deprivation. Otherwise they are marked as –. The various semantic groupings under which the different features fall are also listed.

We observe a negative relationship between the “activeness” of an individual in his or her mobile interactions and poverty. For instance, the delay in responding to text has a positive relationship to poverty. Interestingly, the feature of percent initiated interactions (calls) has, again, a positive relationship to poverty, signifying that in Senegal individuals living in more deprived communes are more likely to initiate calls (for request of resources, etc.) than those living in less deprived communes. The mobility patterns of individuals, captured using spatial features such as number of frequent antennas, entropy of antennas, and total number of antennas used by an individual, indicate a negative relationship to poverty. Thus, individuals living in more deprived communes tend to move fewer antennas than those living in less deprived communes. This observation should be viewed cautiously because of sparse antenna density in rural communes.

## GP Regression Model

The following model is assumed to predict poverty for a commune from a single data source (CDR or environment):

$$y_{i}=\beta^{⊤}{\text{𝐱}}_{i}+f\left({\text{𝐱}}_{i}\right)+\epsilon$$ [S1]

where $y_{i}$ is the target poverty value and ${\text{𝐱}}_{i}$ is a vector of independent variables derived from the particular view for the $i^{th}$ commune. Instead of assuming a fixed parametric form for f(), we adopt a nonparametric approach, by assuming a GP prior on f(), with zero mean function, and kernel function k(). The generative process thus becomes:

$$f\left(\text{𝐱}\right)∼GP\left(0,k(\text{𝐱},\text{𝐱}\prime )\right)y_{i}∼N\left({𝜷}^{⊤}{\text{𝐱}}_{i}+f\left({\text{𝐱}}_{i}\right),\sigma_{n}^{2}\right),\forall i$$

A GP is a stochastic process, such that any finite sample generated from this stochastic process is jointly multivariate normal (15).

The posterior distribution of $f\left({\text{𝐱}}_{*}\right)$ at a test input, ${\text{𝐱}}_{*}$, can be computed given a training set of examples, ${\left\{{\text{𝐱}}_{i},f\left({\text{𝐱}}_{i}\right)\right\}}_{i=1}^{N}$. The joint distribution of the training outputs, $f\left({\text{𝐱}}_{1}\right),f\left({\text{𝐱}}_{2}\right),\ldots$, and the test output, $f\left({\text{𝐱}}_{*}\right)$, according to the GP prior is:

$$\left[\begin{array}{c} f\left({\text{𝐱}}_{1}\right)\\ f\left({\text{𝐱}}_{2}\right)\\ \vdots \\ f\left({\text{𝐱}}_{N}\right)\\ f\left({\text{𝐱}}_{*}\right) \end{array}\right]∼N\left(\text{𝟎},\left[\begin{array}{cccc} k\left({\text{𝐱}}_{1},{\text{𝐱}}_{\text{𝟏}}\right) & \ldots & k\left({\text{𝐱}}_{1},{\text{𝐱}}_{\text{𝐍}}\right) & k\left({\text{𝐱}}_{1},{\text{𝐱}}_{*}\right)\\ k\left({\text{𝐱}}_{2},{\text{𝐱}}_{\text{𝟏}}\right) & \ldots & k\left({\text{𝐱}}_{2},{\text{𝐱}}_{\text{𝐍}}\right) & k\left({\text{𝐱}}_{2},{\text{𝐱}}_{*}\right)\\ \vdots & \ddots & \vdots & \vdots \\ k\left({\text{𝐱}}_{N},{\text{𝐱}}_{\text{𝟏}}\right) & \ldots & k\left({\text{𝐱}}_{N},{\text{𝐱}}_{\text{𝐍}}\right) & k\left({\text{𝐱}}_{N},{\text{𝐱}}_{*}\right)\\ k\left({\text{𝐱}}_{*},{\text{𝐱}}_{\text{𝟏}}\right) & \ldots & k\left({\text{𝐱}}_{*},{\text{𝐱}}_{\text{𝐍}}\right) & k\left({\text{𝐱}}_{*},{\text{𝐱}}_{*}\right) \end{array}\right]\right)$$

For notational simplicity, let K denote a N×N matrix that contains the kernel computation on each pair of training inputs—that is, $K\left[i,j\right]=k\left({\text{𝐱}}_{i},{\text{𝐱}}_{j}\right)$—**k** be a vector of the kernel computation between each training input and the test input—that is, $\text{𝐤}\left[i\right]=k\left({\text{𝐱}}_{i},{\text{𝐱}}_{*}\right)$—and $k_{*}$ be the self-covariance for **x**—that is, $k_{*}=k\left({\text{𝐱}}_{*},{\text{𝐱}}_{*}\right)$. Moreover, let **f** be a N×1 vector, such that $\text{𝐟}\left[i\right]=f\left({\text{𝐱}}_{i}\right)$. The above equation can be written as:

$$\left[\begin{array}{c} \text{𝐟}\\ f\left({\text{𝐱}}_{*}\right) \end{array}\right]∼N\left(\text{𝟎},\left[\begin{array}{cc} K & \text{𝐤}\\ {\text{𝐤}}^{⊤} & k_{*} \end{array}\right]\right)$$

Since **f** and $f\left({\text{𝐱}}_{*}\right)$ are jointly Gaussian, one can make use of the well-known Gaussian identity (43) for the conditional distribution of $f\left({\text{𝐱}}_{*}\right)$—that is:

$$f\left({\text{𝐱}}_{*}\right)|\text{𝐟}∼N\left({\text{𝐤}}^{⊤}K^{-1}\text{𝐟},k_{*}-{\text{𝐤}}^{⊤}K^{-1}\text{𝐤}\right)$$ [S2]

We assume that the observed poverty for the $i^{th}$ commune, $y_{i}$, is equal to the sum of the linear term, the latent function value, with zero mean GP prior, and an independent and identically distributed Gaussian noise ($∼N\left(0,\sigma_{n}^{2}\right)$). Thus, the prior on the observed data will be:

$$𝔼\left[y_{i}\right]=\beta^{⊤}{\text{𝐱}}_{i}\;\text{cov}\left[y_{i},y_{j}\right]=k\left({\text{𝐱}}_{i},{\text{𝐱}}_{j}\right)+\delta_{ij}\sigma_{n}^{2}$$

where $\delta_{ij}$ is the Kronecker delta, such that $\delta_{ij}=1$, if (i=j), and 0 otherwise. For the entire training dataset:

$$𝔼\left[\text{𝐲}\right]=𝐛\;\text{cov}\left[\text{𝐲}\right]=K+\sigma_{n}^{2}I$$

where **b** is a N length vector, such that $\text{𝐛}\left[i\right]=\beta^{⊤}{\text{𝐱}}_{i}$, and I is the N×N identity matrix. The joint distribution of **y** and $f\left({\text{𝐱}}_{*}\right)$ can be written as:

$$\left[\begin{array}{c} \text{𝐲}\\ f\left({\text{𝐱}}_{*}\right) \end{array}\right]∼N\left(\left[\begin{array}{c} \text{𝐛}\\ \beta^{⊤}{\text{𝐱}}_{*} \end{array}\right],\left[\begin{array}{cc} K+\sigma_{N}^{2}I & k\\ k^{⊤} & k_{*} \end{array}\right]\right)$$

Using the conditional Gaussian result, similar to Eq. **2**, and noting the relation between $y_{*}$ and $f\left({\text{𝐱}}_{*}\right)$ from Eq. **1**, the conditional distribution for the prediction $y_{*}$ becomes:

$$𝔼\left[y_{*}\right]=\beta^{⊤}{\text{𝐱}}_{*}+{\text{𝐤}}^{⊤}{\left(K+\sigma_{n}^{2}I\right)}^{-1}\left(\text{𝐲}-\text{𝐛}\right)\;\text{var}\left[y_{*}\right]=k_{*}-{\text{𝐤}}^{⊤}{\left(K+\sigma_{n}^{2}I\right)}^{-1}\text{𝐤}+\sigma_{n}^{2}$$

## Estimating Moments of a Mixture Distribution

Let random variable y represent a mixture of two unimodal normal distributions, $y_{1}∼N\left(\mu_{1},\sigma_{1}^{2}\right)$ and $y_{2}∼N\left(\mu_{2},\sigma_{2}^{2}\right)$ and mixing probabilities $w_{1}$ and $w_{2}$, such that $w_{1}+w_{2}=1$—that is:

$$y=w_{1}y_{1}+w_{2}y_{2}$$

Any moment of y can be computed as (44):

$$𝔼\left[y^{k}\right]=w_{1}𝔼\left[y_{1}^{k}\right]+w_{2}𝔼\left[y_{2}^{k}\right]$$

which directly gives:

$$𝔼\left[y\right]=w_{1}\mu_{1}+w_{2}\mu_{2}$$

The expression for the variance of y can be derived as follows:

$$\text{var}\left[y\right]=𝔼\left[y^{2}\right]-{\left(𝔼\left[y\right]\right)}^{2}=w_{1}𝔼\left[y_{1}^{2}\right]+w_{2}𝔼\left[y_{2}^{2}\right]-{\left(w_{1}\mu_{1}+w_{2}\mu_{2}\right)}^{2}=w_{1}\left(\text{var}\left[y_{1}\right]+\mu_{1}^{2}\right)+w_{2}\left(\text{var}\left[y_{1}\right]+\mu_{1}^{2}\right)-{\left(w_{1}\mu_{1}+w_{2}\mu_{2}\right)}^{2}=w_{1}\sigma_{1}^{2}+w_{2}\sigma_{2}^{2}+w_{1}\mu_{1}^{2}+w_{2}\mu_{2}^{2}-w_{1}^{2}\mu_{1}^{2}-w_{2}^{2}\mu_{2}^{2}-2w_{1}w_{2}\mu_{1}\mu_{2}=w_{1}\sigma_{1}^{2}+w_{2}\sigma_{2}^{2}+w_{1}w_{2}\mu_{1}^{2}+w_{1}w_{2}\mu_{1}^{2}-2w_{1}w_{2}\mu_{1}\mu_{2}=w_{1}\sigma_{1}^{2}+w_{2}\sigma_{2}^{2}+w_{1}w_{2}{\left(\mu_{1}-\mu_{2}\right)}^{2}$$

The last result makes use of the fact that $w_{1}+w_{2}=1$.

## Interpretation of Weights—Along the Dimensions of Poverty

This section complements *Dimensions of Poverty—Interpretation of Weights* and refers to Fig. S2. These interpretations are given for information purposes and are by no means indicators of causality.

Several features related to the presence of water in the commune (water bodies, water, mudflat soil, hydro-morphic soil, and elevation) are positively correlated with water deprivation, although the opposite is observed for the other dimensions. One interpretation is that natural nonportable water would be used for drinking in these areas. On the other hand, access to water is needed for irrigated agriculture, watering livestock, or fishing, all of which can increase income and life quality, which explains the negative relationship for the other dimensions. Interestingly, the distance to a water tower is not quite correlated with this deprivation. Alternatively, the proximity to a water forage would have probably been a more interesting feature.

The food security (access) features (like distance to main roads and urban centers) are also prominent, stressing their importance for development. Millet price has a mixed behavior. Depending on the dimensions, its coefficient is sometimes positive and sometimes negative, without any evident explanation.

The effect of temperature is clear. The higher the maximum temperature and the range, the higher the poverty. Temperature plays a role in crop growth, but it also impacts the environment quality of the people who live in warm (and cold during the night) areas.

The effect of precipitation is less obvious. The amount and the period of rainfall affects the availability of water, which is the main limiting factor in Sahel for crop and forage production. The precipitation seasonality, described by the period during which the water is available, and the precipitation of the warmest quarter (critical period) are logically negatively correlated with poverty. However, the annual precipitation and the precipitation of the wettest month and quarter have a positive coefficient (except for education deprivation). In other words, the more it rains in an area, the poorer it is. The intuition would have been that it was the opposite. But looking more closely, it appears that several features related to agriculture (groundnut production, cassava production, rain-fed croplands) show the same patterns. We interpret that these features define a suitable environment for agricultural areas, which itself is linked to the presence of a rural community tending more to poverty than the urban population.

## Discussion

The technological advances over the past decade have led to building of communication devices (like phones) and sensors (like satellites and weather and ground sensors) that produce and store a myriad of data. In this work, we show how these novel sources of data, which are characterized by their volume, variety, and associated uncertainty, can be used to generate accurate poverty maps.

We outline several challenges that lie in establishing relationships between auxiliary data sources (that are not collected to directly measure socioeconomic deprivations) and poverty. The first challenge occurs due to the varying spatial granularity at which the different datasets are available; this requires an aggregation mechanism to link them. CDR data are available for each subscriber, while environmental data have mixed spatial resolution, from very accurate vector data to low-resolution satellite imagery (1 km). On the other hand, census data are available for individuals or households, depending on the response variable. However, given that the individual information is anonymized for both CDRs and census data, there is no obvious way to link the records across these two datasets. In this work, we localize the individuals and/or households to their respective communes, or urban centers, by using their census information (details in *Materials and Methods*). This lets us calculate the commune-level deprivations. For CDRs, the individuals are localized to their home antennas based on their most frequent night location. The CDR and environmental data are aggregated to commune levels. Though we have taken a commune as the level of aggregation, the framework allows for the same analysis at even finer spatial resolutions.

A key concern associated with using CDR data for population-level analyses is the selection bias arising from mobile phone ownership. In Senegal, however, there were 92.93 mobile phone subscriptions per 100 inhabitants in 2013, which implies that most of the population owns cell phones (24). The second challenge is the bias arising when using data from only one provider. However, the provider of the data used here, Sonatel, had nearly 62% of the cell phone market in 2013 (25). The third concern is that some demographic subgroups like children and the ultra poor are left out by the analysis while only using CDR data. Also, results may be biased toward urban regions, rather than rural regions, because of factors like lack of electricity in rural areas.

Here, we used two distinct types of environment data. The first type includes static natural/physical environment variables (like elevation, soil types, etc.) or long-term dynamic phenomena (like climate). The second type includes human-induced aspects, like urban areas, roads, access to facilities, and so forth. Though the natural environment acts as a constraint in designing poverty eradication plans, effective policies and sustainable approaches should be made an integral part of policy planning. Environmental features derived from satellite images (nighttime lights, NDVI, etc.) have the potential to be computed in near real-time to monitor the impact of shocks such as natural hazards, armed conflicts, or crop pests that can rapidly cause serious deprivations. However, for reliability, these variables need to be aggregated for a longer period, typically at an annual level for nighttime lights and for the growing season for NDVI. OpenStreetMap (OSM) data, which are used to map facilities and roads, are crowd-sourced and therefore have the (theoretical) potential to be updated in near real time. This capability could be limited in African countries. Due to the above constraints, 1 y is probably the relevant period for consistent monitoring of poverty with our method (compare with 3–5 y for a detailed and costly census).

Another challenge is the ease of availability of data. Environmental datasets are available to researchers for free and typically have no privacy constraints, especially at the resolution at which it is analyzed here. CDR data are collected by commercial telecommunication entities and might suffer from lack of accessibility to researchers due to sharing constraints between different organizations. However, our methodology requires no raw data to be shared between different data-owning entities; only the output predictions from each individual model and the associated uncertainties are combined at the final step.

An important consideration is the number of features extracted from the data. Recent work (20) has used four features—namely, call volume and mobile ownership per capita, nightlights, and population density—to estimate the MPI of sectors in Rwanda using a linear regression model. As a baseline for our model, we used the same features and model to predict MPI values at the commune level in Senegal. A spatially cross-validated Pearson’s correlation of 0.84 was achieved with a significant *P* value (<0.0001) (see Table S8 for comparison). Although less features provide computational tractability of analysis, they offer no insight into other features that could be useful in understanding poverty. Also, linear models are limited in their ability by the linearity assumption and sensitivity to outliers.

**Table S8.** Comparative table showing how our model performs compared with only nightlights and a previous work (used as a baseline) using only four features—namely, call volume and mobile ownership per capita, nightlights, and population density

Data source	Model	Results, Pearson’s *r*
Nightlights	Linear regression	0.39
(20)	Linear regression	0.84
Our model	GP regression	0.91

**Fig. 4.**
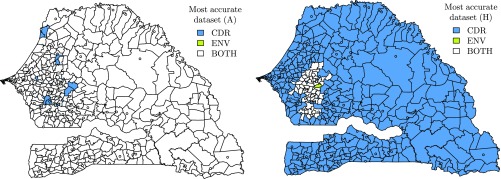
The uncertainty associated with each dataset evidenced by the most accurate one (denoted as CDR and ENV) for the average intensity of poverty (A) (*Left*) and prediction of the headcountof poverty (H) (*Right*).

An important advantage of our GPR model is that each predicted poverty value is associated with an uncertainty (generated by the model). This highlights the strength of confidence in the predictions and can be used as guidance by policy makers. Comparing these source-specific uncertainties can reveal which data hold a better signal for a specific prediction (see Fig. 4). We note that for predicting A, the predictions of CDRs and environment data are comparable for most of the communes. For predicting H, CDRs perform with lower uncertainties than environmental data. These variations may be attributed to multiple reasons, including resolution and concurrency of data, demographics and mobile penetration of the cellular provider, and spatial heterogeneity of poverty deprivations.

Though we have discussed the methodology for predictions at the commune level, our predictions of MPI and associated dimensions can be successfully aggregated to coarser administrative units, if needed, for policy planning. Since we use global MPI as the poverty index, its limitations, as noted by global MPI researchers (26), are applicable to our study as well. In particular, global MPI does not include characteristics such as parents’ education, social norms and beliefs, empowerment, etc.

Additionally, it will be interesting to see how well this methodology can be used to predict other indicators of deprivation and inequality, like the GINI index, at the microregional level. Apart from being useful in producing interim statistics in between long cycles of census and surveys, such methodology can also be extended to places of conflict or remote areas that are difficult to reach by census takers.

**Fig. S4.**
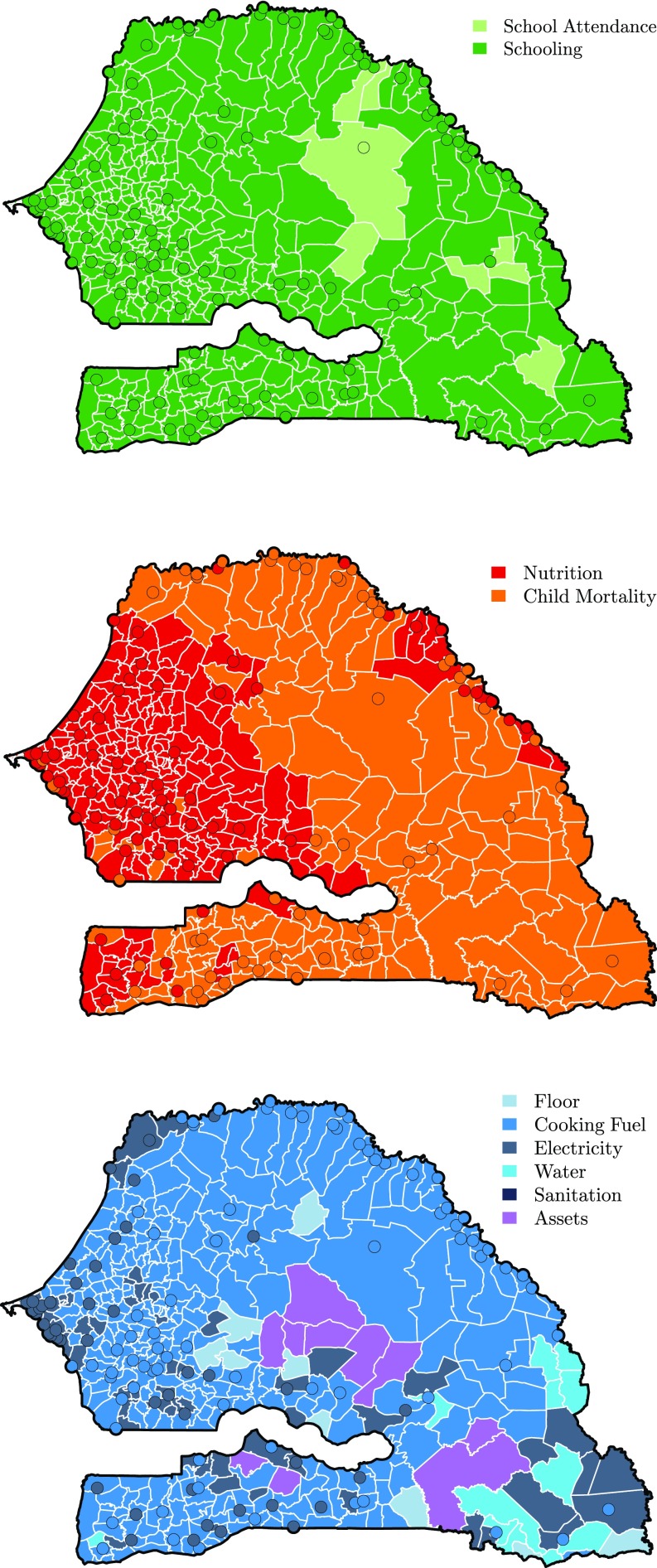
The highest deprivation by commune as predicted by our model for each dimension of global MPI (from top to bottom: education, health, and standard of living).

As described in the results, the interpretation of the model coefficients provides some insights on the dimensions of MPI. However, due to the number of variables, this interpretation is still complex and not necessarily straightforward for policy intervention. Conversely, the MPI dimensions are well-known factors for which policy planning is feasible (26). As an illustration, Fig. S4 shows the highest predicted deprivation for each commune within each dimension.

Lastly, though GPR model uncertainty is impacted by the bias and inaccuracy of each data source (quality of soil type map, interpolation of climatic data, missing facilities, mobile operator’s market share), a higher resolution and accuracy of the input data should benefit the modeling relevance and quality.

## Materials and Methods

### Target Country.

Senegal is a sub-Saharan country that ranks 170 on the Human Development Index with a score of 0.466 and a population of 14.5 million (with 43.5% urban population) (27). As one of the poorest countries in the world, it has 52% of the population living in multidimensional poverty (27). On the other hand, there are 98.8 mobile phone subscriptions per 100 people (24). Senegal is composed of 14 coarsest administrative units called regions, which are further divided into 45 administrative units called departments. The finest level of administrative units is called a commune. There are 552 communes (121 as urban centers and 431 rural) (Fig. 1).

### Data Sources.

#### CDRs.

A CDR consists of an identifier with the caller and callee, the antenna location of the caller, the time of the call, duration of the class, and a flag indicating if the record is a text or a call. A CDR is generated each time a call or text is placed. The data belong to the subscribers of Sonatel, Orange, which is the dominant telecom provider in Senegal. The data are anonymized and span a period from January 1 to December 31, 2013. They contain more than 9.54 million unique aliased mobile phone subscribers. The population of Senegal in 2013 was 14.13 million. Additionally, the geographical coordinates of the mobile antennas are known, and shown in Fig. 1.

#### Environmental Features.

Based on literature, several environmental features that may have a relationship with poverty have been explored (see Table S1). They are either based on Geographical Information System (GIS), Earth Observation data, or weather stations.

#### Census.

The Agence Nationale de la Statistique et de la Demographie (ANSD), which is the National Statistics Office of Senegal, provided us with a 10% sample of the 2013 census [called RGPHAE (Recensement General de la Population de l’Habitat de l’Agriculture et de l’Elevage)]. The data are evenly sampled across the entire population of Senegal and are from 1.4 million individuals, spread across 150,000 households, characterizing information related to demographic statistics (mortality, fertility, migration, literacy, occupation, etc.), along with habitat features, such as type of roof, floor, access to drinking water, sanitation, and agriculture practices. The advantage of the census is that it represents important national statistics at the level of individuals. Brief statistics of the data sources are given in Table 1.

The mobile phone data used in this study can be obtained for replication purposes by contacting Zbigniew Smoreda (zbigniew.smoreda@orange.com).

### Feature Extraction.

#### CDRs.

We have access to more than 11 billion mobile phone transactions involving calls and texts for a year in Senegal. Each time a call or text is placed, it is logged as a transaction. Missed, forwarded, and other *undelivered* calls were removed from the logs.

To extract important features that quantify the mobile use pattern of a subscriber, we focus on well-studied metrics capturing the individualistic, spatial, and temporal patterns of the subscriber (28–30). The individual aspects quantify the typical use pattern of an individual. Some of the metrics that belong to this category are the number of active days, the number of contacts, the average call duration, percent nocturnal, and so forth. Spatial metrics are the ones that quantify the typical movement pattern of an individual. Examples of spatial metrics for a subscriber include radius of gyration, entropy of antennas, and so forth. There are 43 core features (briefly described in Table S2), extracted using the *Bandicoot* toolbox (31). All features were calculated at monthly granularity capturing the temporal aspect of a subscriber, resulting in 43×12 CDR-based features.

The second step is to localize each subscriber, i, to his or her home antenna. A home antenna, $h_{i}$, is calculated as one from where the subscriber makes the most nocturnal calls (from 7 PM to 7 AM) during each month. We filtered out individuals who made less than five calls during each month and who were not active for at least half of the year within the range of their home antennas. This ensures that individuals are reliably allocated to their home antennas. After the filtering step, the sample contained 6.19 million individuals (65% of the original subscriber population).

We then computed the average feature value for each antenna site by computing the average of the feature values for all individuals who consider that antenna as their home:

$$m_{a}^{\left(f\right)}=\frac{1}{N_{a}}\sum_{i:h_{i}=a}m_{i}^{\left(f\right)}$$ [9]

where $m_{i}^{\left(f\right)}$ is the *f*th feature value.

Finally, we compute the feature value for each commune as the weighted average of all antennas whose voronoi polygon intersects with the commune boundary as:

$$m_{c}^{\left(f\right)}=\frac{1}{\sum w_{c,a}}\sum w_{c,a}m_{a}^{\left(f\right)}$$ [10]

The weight $w_{c,a}$ is the ratio $\frac{Area\left(c\cap a\right)}{Area\left(a\right)}$, which is a measure of how much of the voronoi cell for antenna a falls within the boundary of commune c. To study how well the Voronoi-based approach has performed in assigning people to their communes, we study the correlation of the commune population estimated by this approach and that calculated from census. The Pearson’s correlation is reported as 0.85 with a *P* value of <0.00001, thus ensuring the validity of our approach.

#### Environmental features.

In this study, we focus on three broad categories of environmental features: food security (divided into the availability and access components), economic activity, and access to services (see Table S1). These three categories cover most of the features that have been shown to be significantly related to poverty in the literature (see Table S3).

Food security is mainly described by agrometeorological measurements (temperature, precipitation, slope, elevation, soil type) that drive agricultural production (crop production), one of the most important inputs, along with livestock and fishing, of food availability in the country. On the other hand, access to staple food can be approximated by the average millet prices observed in the markets (retail prices in 56 local markets). Millet serves as the main local staple food crop in the country, making it a potentially good indicator of poverty. In addition, proximity to main road and urban centers was also computed to describe the connectivity to major markets.

The economic activity corresponds to the intensity of urbanization. Among the studied features, the nighttime lights are the most frequently used to describe poverty using remote-sensing data (20). Moreover, a clear link between household wealth and the level of night light emissions has been shown before (32). The underlying hypothesis is that economic activity and urbanization are strong indicators of living standards.

Finally, the access to services can help to predict some of the individual indicators of poverty. In particular, the proximity to school, water towers, and hospitals can be used to determine the deprivation in education, water, and health, respectively.

The raw environmental data are available either in raster grid (at different spatial resolutions) or in vector format. As a first step, all vector data were converted into raster grid format. Then, all data layers were resampled (using the nearest neighbor approach) at a spatial resolution of 100 m. Pixel values falling within each commune’s boundary were averaged to give a unique value for that commune.

All environmental data are available at high spatial resolution, with the exception of crop production and millet prices (see Table S1 for the data sources). Millet prices were available in 56 local markets, potentially missing some of the local heterogeneity. Production estimation features were derived from the Direction de l’Analyze, de la Prévision et des Statistiques Agricoles (DAPSA) database. The granularity of these features is at the department level. Cultivated areas were masked using the 2005 1:100,000 Scale Senegal Land Cover Map produced by the Global Land Cover Network based on the GlobCover 2005 map (33), which is the most accurate map for Senegal (34). Since reliable information on the spatial distribution of each crop is unavailable, we made an assumption that they were grown evenly within the cultivated areas of a specific department. Therefore, the production of a specific department was distributed evenly among all of the 100-m pixels that fell within the cropland of this department. This raster was then used to aggregate the production estimations by communes.

The Normalized Difference Vegetation Index (NDVI) is used as a proxy of potential agricultural production within a department. The NDVI, defined as the difference between near-infrared and red reflectances normalized by the sum of the two parameters, is a useful yield proxy in regions where water or soil fertility are the main limiting factors, such as Sahel (35, 36). For each pixel within cultivated areas, NDVI values above 0.2 during the growing season (July to November) were integrated (TNDVI), which limited the contribution of bare soil to the signal.

### Model Training.

The unknown parameters of each source-specific model in Eq. **1** are as follows: the parameter β of the linear component, the hyperparameters of the kernel function $\ell ,\ell_{s},\sigma_{f}^{2}$, and the variance of the error term $\sigma_{n}^{2}$. These are estimated by maximizing the marginalized likelihood of the target poverty values in the training data **y**. The marginalized likelihood is obtained by taking the integral of the likelihood times the prior:

p(𝐲|𝐗)=∫p(𝐲|f,X)p(𝐟|𝐱)d𝐟 [11]

where the matrix **X** contains the training input vectors as rows and **f** is a vector containing the latent function values for the inputs in **X**. The GP prior means that p(𝐟|𝐗)∼N(𝟎,K) and the likelihood is a Gaussian—that is, $p\left(\text{𝐲}|\text{𝐟},\text{𝐗}\right)∼N\left(\text{𝐗}𝜷+\text{𝐟},\sigma_{n}^{2}I\right)$. The integration of Eq. **11** yields the following marginalized log likelihood (15) of the training data:

$$\log p\left(\text{𝐲}|\text{𝐗}\right)=-\frac{1}{2}{\left(\text{𝐲}-\text{𝐗}\beta \right)}^{⊤}{\left(K+\sigma_{n}^{2}I\right)}^{-1}\left(\text{𝐲}-\text{𝐗}\beta \right)-\frac{1}{2}\log|K+\sigma_{n}^{2}I|-\frac{N}{2}\log2\pi$$ [12]

where N is the number of training examples.

To regularize the coefficients in β, we apply elastic net regularization on the marginalized log likelihood to obtain the following objective function:

$$J\left(\beta ,\ell ,\ell_{s},\sigma_{n}^{2},\sigma_{f}^{2}\right)=\log p\left(\text{𝐲}|\text{𝐗}\right)-\left(\alpha \lambda ∥\beta {∥}_{2}^{2}+\left(1-\alpha \right)\lambda |\beta |\right)$$ [13]

The function J is maximized to estimate the hyperparameters using conjugate gradient descent (37).

All codes used to replicate the results can be obtained by writing to the corresponding author.

### Regularization.

Regularization techniques, such as those used in Lasso (38) or Ridge regression (39), are often used to improve model performance, especially when the data contain several irrelevant features. The $L_{2}$ penalty, imposed by Ridge regression, ensures shrinkage of regression coefficients to avoid overfitting. On the other hand, the $L_{1}$ penalty imposed by Lasso forces the coefficients to be sparse, thereby providing feature selection. However, neither of the two regularization methods have been found to universally dominate the other (38). For instance, in the presence of groups of correlated features, Lasso tends to select only one feature within each group, which leads to poor interpretability of the estimated coefficients. Elastic net regularization (17) is a weighted addition of $L_{1}$ and $L_{2}$ penalties and combines the strengths of both Lasso and Ridge regression. It is known to select a greater number of influential features than Lasso and has a lower false-positive rate than ridge regression.

We used elastic net regularization to penalize complexity of the solution and to avoid overfitting on the limited training dataset. The elastic net penalty is computed as:

$$\alpha \lambda {∥\beta ∥}_{2}^{2}+\left(1-\alpha \right)\lambda \left|\beta \right|$$ [14]

Our empirical results show that elastic net regularization results in better prediction accuracy, compared with ordinary least squares, Ridge, and Lasso regression.

### Model Validation.

This section details the steps followed to validate our model, namely creating commune-level poverty statistics from census data and methodology for spatial CV.

#### Creating commune poverty statistics from census.

The 10% sample of the 2013 RGPHAE census, used here, has survey responses for 150,000 households and 1.4 million individuals pertaining to their socioeconomic indicators (literacy, birth and death in the family, etc.) and habitat (type of house, access to electricity and drinking water, etc.). Some survey responses are individualistic (like literacy and profession), while others are associated with the entire household (like type of roof, sanitation, electricity).

The first step is to assign the individuals to their respective households using information from the fields in the census. The second step is to calculate per-household deprivations in the poverty indicators of interest. Global MPI computation (26) requires deprivations along three dimensions (with 10 indicators)—namely, health (child mortality, nutrition), education (child school attendance, years of schooling), and standard of living (electricity, sanitation, drinking water, flooring, cooking fuel, assets).

We follow the procedure similar to the widely used Alkire–Foster methodology for computing MPI (40). First, we create a deprivation vector $depvec_{i,d}$ corresponding to each household i in poverty indicators d=1,…,D. Each vector entry is either 1 if $y_{i,d}$ $\leq z_{d}$, where $y_{i,d}$ is the achievement of household *i* in indicator d and $z_{d}$ is the cutoff score in indicator *d*, or 0 otherwise. A value of 0 for $depvec_{i,d}$ implies nondeprivation of the household in that particular indicator. For the values of cutoff scores for different indicators, see Table S7. We aggregate all households that are deprived in a particular indicator, for each commune, and divide by the total number of households in that commune. This score gives the proportion of households deprived in a particular indicator within a commune.

Since MPI is a multiplicative combination of H and A—that is, MPI=H×A—we first calculate H and A. For H, we introduce a weight, $w_{d}$, for each indicator d. For each household, we compute a weighted deprivation score, $c_{i}=\sum_{d=1}^{D}w_{d}depvec_{i,d}$. The weights $w_{d}$ are assigned as follows. The education- and health-related indicators are given a weight of $\frac{1}{6}$, while each of the six standard of living indicators are given a weight of $\frac{1}{18}$. Thus, each dimension has a weight of $\frac{1}{3}$.

$H_{j}$, which is the relative headcount of poor households in commune j, is calculated as:

$$H_{j}=\frac{1}{N_{j}}\sum_{i=1}^{N_{j}}I\left(c_{i}>\theta \right)$$ [15]

where θ is a cutoff, whose higher values mean a higher cutoff for household achievement, and $I\left(c_{i}>\theta \right)$ is the indicator function. $N_{j}$ is equal to the total number of households in the *j*th commune.

To calculate A, we count only the poor households, and their deprivations, as follows:

$$A_{j}=\frac{1}{\sum_{i=1}^{N_{j}}I\left(c_{i}>\theta \right)}\sum_{i=1}^{N_{j}}I\left(c_{i}>\theta \right)*c_{i}$$ [16]

The value of threshold θ is taken as 0.3. We varied θ from 0.2 to 0.75, and the H and A values obtained in each run were correlated with region-level H and A, available from University of Oxford’s MPI calculation [Oxford Poverty & Human Development Initiative (OPHI)]. The results were stable and peaked at 0.3, which is also the threshold value taken by OPHI for its calculations.

#### Spatial CV.

To measure the extrapolation capacity of the model on out-of-sample data, spatial CV techniques, where training and evaluation sets are sampled from geographically distinct regions, are more robust (18, 41). The following spatial CV strategy was adopted: For each CV run, we first randomly sampled a region r from the set of 14 regions and then randomly sampled a commune c belonging to r. All communes that lie within distance d of the commune c are included in the training dataset. The remaining communes are included in the evaluation dataset.

This strategy ensures that communes from all regions of Senegal are represented in the training and evaluation datasets during CV. To ensure that the training dataset has enough examples, we forced at least 40% of the communes (225) to be included in the training dataset. To achieve this, d is initially set to 100 km and is increased by 50 km until the size of the training dataset meets the threshold. CV is repeated 250 times. We report the mean predictive performance (using Pearson’s and Spearman’s correlation and RMSE values) on the evaluation dataset, along with the SD across multiple runs.

## Understanding Model Uncertainty

The predictive variance associated with the GP model, as calculated using Eq. **4**, indicates the model uncertainty for a test target. The variance does not depend on the observed target values, only on the inputs. The variance at a given test commune is directly related to how many similar communes (in terms of the CDR, environmental, and spatial features) are available in the training data. For instance, if the predictive variance is high for a given test commune, it would mean that the relative density of the training feature vectors in proximity of the feature vector corresponding to the test commune is low, and hence the GP model will yield a higher predictive variance. This could explain the higher variance for MPI predictions observed for rural communes compared with urban communes.
